# Obstructive Jaundice as Initial Presentation of Multiple Myeloma: Case Presentation and Literature Review

**DOI:** 10.1155/2015/686210

**Published:** 2015-06-21

**Authors:** Yasir Khan, Iyad Mansour, Eric Ong, Manish Shrestha

**Affiliations:** University of Arizona Internal Medicine Residency, 1501 N. Campbell Avenue, Tucson, AZ 85724, USA

## Abstract

Multiple myeloma is a malignant plasma-cell disorder that primarily involves the bone marrow, but extramedullary involvement is becoming increasingly common (Bladé et al., 2012) both at initial presentation and follow-up. Most common initial presentations for multiple myeloma include generalized fatigue, renal insufficiency, bone pain, and recurrent bacterial infections. We present a case of a healthy 55-year-old man that presented to the emergency department with a three-week history of anorexia and jaundice without any past medical history. Patient's initial labs were significant for hyperbilirubinemia and elevated liver function enzymes (AST, ALT, ALP, and GGT). Additional laboratory workup was significant for mild hypercalcemia and increased protein gap. MRI and ERCP suggested primary sclerosing cholangitis but were not diagnostic. Liver biopsy illustrated plasma-cell infiltration and bone marrow biopsy diagnosed multiple myeloma with extramedullary disease. Patient was started on dexamethasone, bortezomib, and cyclophosphamide, but, despite this aggressive regimen, the patient continued to decline. We take this opportunity to present this atypical presentation of a common hematological malignancy and review the associated literature.

## 1. Introduction

Multiple myeloma accounts for one percent of human cancers, almost two percent of cancer deaths, and 12–15% of all hematological cancers [1]. It is a neoplastic plasma-cell disorder that involves clonal proliferation of malignant plasma cells in bone marrow, monoclonal protein in the blood or urine, and involved organs. Myeloma is thought to arise from monoclonal gammopathy of undetermined clinical significance that progresses to smoldering myeloma and, finally, to symptomatic myeloma [1–3]. Genetic abnormalities change the expression of adhesion molecules on myeloma cells that allows malignant cells to escape regulatory mechanisms and proliferate autonomously [1, 2, 4].

The diagnosis requires presence of 30% monoclonal bone marrow plasma cells and most common initial presentations include anemia (73%), bony lesions (80%), and renal impairment (20–40%) [1]. Although multiple myeloma is a malignancy that predominantly affects bone marrow and bone, involvement of extraosseous tissues like spleen and liver is a relatively frequent postmortem finding [4–6]. The reported incidence of liver involvement in myeloma has been reported to be in the range of 26% to 46% in autopsy studies [7]. Nevertheless, the incidence of premortem clinical manifestations like hepatomegaly or ascites is much lower. Jaundice is an exceptionally rare way of presentation for myeloma and can be due to several causes: postobstructive to infiltration [8–10]. We report a case of a 55-year-old man who presented with jaundice and abdominal pain as the initial clinical manifestations of multiple myeloma.

## 2. Case Presentation

A 55-year-old man with no significant past medical history presented with a three-week history of jaundice, right upper quadrant abdominal pain, and bloody stools. He also complained of generalized fatigue and anorexia for the previous two months. He denied history of heavy alcohol use, liver disease, or inflammatory bowel disease. He also denied history of allergies and was not on any medication at home. On physical examination he was afebrile and icteric, but there were no other stigmata of chronic liver disease. Cardiovascular, respiratory, and abdominal examinations were normal. Laboratory studies showed white blood cell count of 7.4 × 10^9^/L with plasma cells noted on the peripheral blood smear, hemoglobin level of 13.1 g/dL, and platelet count of 95 × 10^9^/L, total bilirubin of 10 mg/dL, alkaline phosphatase (ALP) of 218 IU/L, aspartate aminotransferase (AST) of 96 IU/L, and alanine aminotransferase (ALT) of 47 IU/L. His total protein was 7.7 g/dL, albumin level was 2.4 g/dL, and erythrocyte sedimentation rate (ESR) was 82 mm/hour. Prothrombin time (14.1 sec) and INR (1.1) were normal. Serum creatinine was also normal (0.9 mg/dL), but his corrected calcium was elevated at 11.2 mg/dL. HIV and hepatitis A, B, and C antibodies and autoimmune antibodies were negative.

Chest radiography did not demonstrate any focal pulmonary or bony abnormalities. Abdominal ultrasound was significant for hepatosplenomegaly, contracted gallbladder, and hepatic steatosis. Due to patient's persistent increase in total bilirubin, abdominal MRI was done to evaluate hepatobiliary pathology. Abdominal MRI did not reveal any sign of hepatocellular carcinoma but suggested a chronic intrinsic liver disease with possible sclerosing cholangitic etiology due to presence of focal segmental dilatation of intrahepatic bile ducts at the junction of segments IVa and VIII and beading of the right intrahepatic bile duct (Figure 4).

Subsequently, the patient underwent esophagogastroduodenoscopy (EGD), endoscopic retrograde cholangiopancreatography (ERCP), and colonoscopy to rule out primary sclerosing cholangitis and inflammatory bowel disease. EGD revealed Grade I esophageal varices with normal gastric and duodenal mucosa. ERCP was significant for small intrahepatic ducts with pruning without any obvious beading or ductal stones/debris (Figure 5). Furthermore, colonoscopy was negative for any sign of inflammatory bowel disease.

Lastly, the patient underwent liver biopsy which identified neoplastic plasma cells filling the sinusoids of the liver and a bone marrow biopsy that was diagnostic for multiple myeloma (90% kappa light chain restricted plasma cells with 95% cellularity). Serum protein electrophoresis showed a monoclonal hypergammaglobulinemia. Immunoglobulin quantification was IgA 2112 mg/dL, IgG 225 mg/dL, and IgM 21 mg/dL. Flow cytometry revealed predominantly kappa light chains (11.1) in serum along with lambda light chain of 0.62, kappa/lambda ratio of 17.9, urine M-spike of 2.3, LDH 725, and B2-microglobulin 7.54. FISH analysis was significant for gain of 1q21, t(4; 14), (p16; q32), gain of 15q22, and 17p13 deletion. His disease stage was III according to the International Staging System for multiple myeloma. Upon diagnosis, the patient was started on dexamethasone, bortezomib, and cyclophosphamide; however, his condition continued to deteriorate rapidly with persistent rise in bilirubin, ALP, AST, and ALT. Unfortunately, after three cycles of chemotherapy the patient developed septic shock with encephalopathy and died two weeks later.

## 3. Discussion

Patients with multiple myeloma present mostly with complaints of generalized fatigue, recurrent bacterial infection, weight loss, hematuria, or back pain but rarely of jaundice [1, 11]. The neoplastic plasma cells in multiple myeloma produce monoclonal light chains that frequently deposit in kidney, heart, peripheral nerves, and liver [4]. Although liver is the second most common organ after kidney to be affected by light chains, most of the patients remain asymptomatic and have mild elevations of liver enzymes early in the disease process [6, 12].

Myeloma presenting as jaundice has been reported in literature but the majority of these cases have been due to hepatic amyloid deposition or extrahepatic biliary tract obstruction secondary to abdominal plasmacytoma or pancreatic head myeloma; few cases of liver dysfunction due to plasma-cell infiltration have been reported [8–10]. Our patient presented with complaints of anorexia and jaundice without any complaints of back/bone pain, hematuria, or renal failure. Diagnosis was not immediately obvious as patient had severe cholestasis with MRI and ERCP findings suggestive of primary sclerosing cholangitis (Figures 4 and 5).

**Figure 1:**
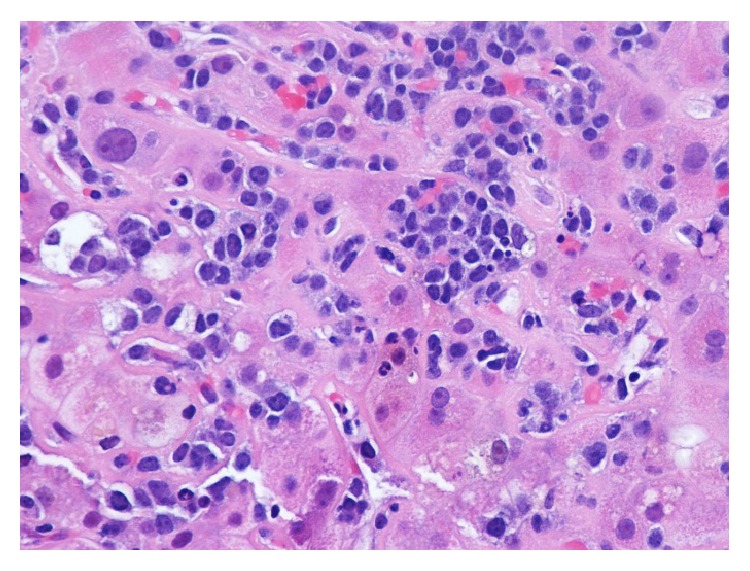
High power image of liver demonstrating sinusoids packed with atypical plasma cells.

**Figure 2 fig2:**
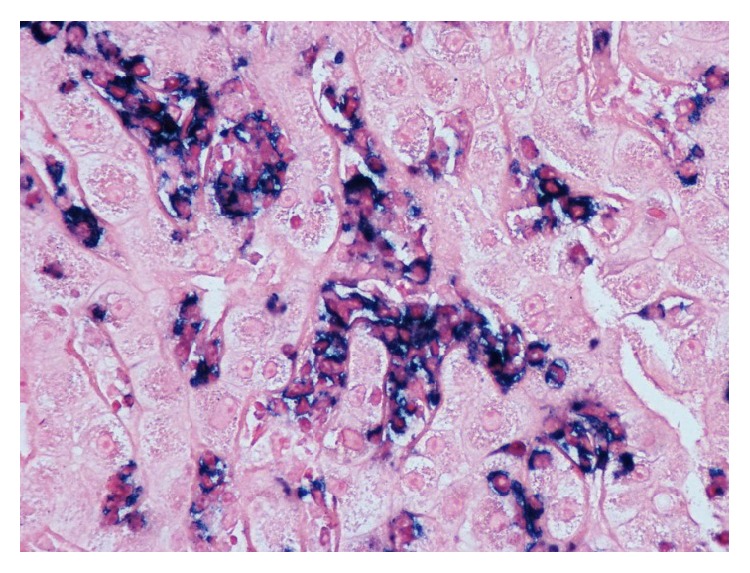
Kappa light chain restriction of the intrasinusoidal plasma cells.

**Figure 3:**
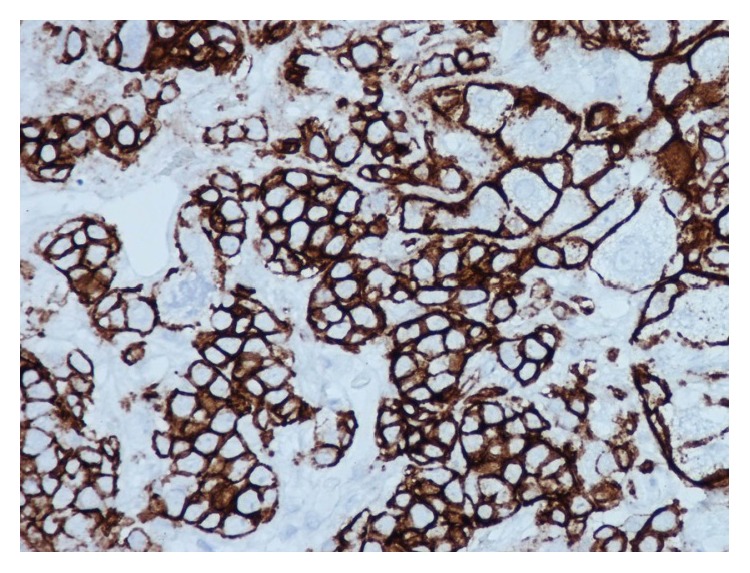
Positive Staining of intrasinusoidal plasma cells for CD138.

**Figure 4:**
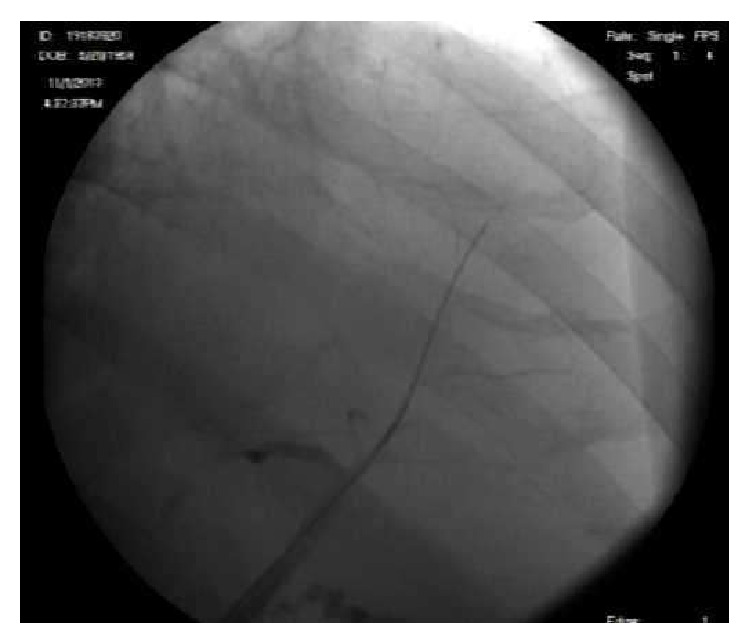
ERCP showing pruning of the biliary tree.

**Figure 5:**
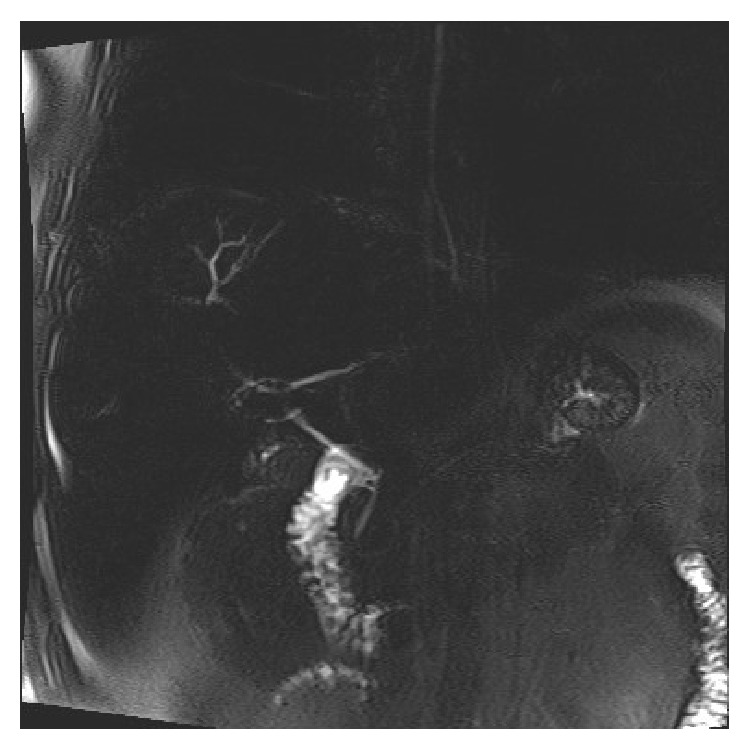
MRCP significant for right intrahepatic beading.

Our case is unique as it highlights a rare presentation for multiple myeloma as obstructive jaundice. Patient denied any symptoms suggestive of multiple myeloma. Admission labs did not reveal anemia or renal insufficiency but were significant for thrombocytopenia, abnormal liver function tests, mild hypercalcemia, and increased protein gap. Abdominal MRI and ERCP were suggestive of sclerosing cholangitic etiology. Thrombocytopenia is a common finding in patients with liver disease and increased protein gap can be seen in several diseases ranging from autoimmune to infectious ones [13]. Furthermore, primary sclerosing cholangitis may manifest with IgM hypergammaglobulinemia [14]. Since ERCP was nondiagnostic, liver biopsy was obtained (Figures 1 [Fig fig2]–3) that led to bone marrow biopsy and, ultimately, to the diagnosis of multiple myeloma. Though the patient was diagnosed in a timely manner, after presentation, chemotherapy course did not alter the course of disease. With extensive extramedullary disease, our patient did not respond favorably to chemotherapy regimen and continued to deteriorate clinically. The patient developed septic shock with encephalopathy and died two weeks later.

Incidence of extramedullary disease in multiple myeloma ranges from 7% to 20% on initial presentation [4, 10, 15, 16]. Most common site for extramedullary disease on diagnosis appears to be skin/soft tissues; however, on follow-up liver seems to be involved more than any other organ [4, 15, 16]. Overall, patients with extramedullary disease tend to have more aggressive disease course, with shorter progression-free survival and overall survival [16]. Once liver failure is established the prognosis is poor and patient progresses rapidly through jaundice, coagulopathy, and encephalopathy [5]. Though current novel agents have increased overall survival of patients to more than ten years, the biologic and genetic features of multiple myeloma play a crucial role in treatment. Patients with high risk chromosomal aberrations and extramedullary disease, like our patient, have poor prognosis. The current diagnostic criteria and staging method for newly diagnosed multiple myeloma patients are listed as below.

*Diagnostic Criteria and Staging System for Multiple Myeloma [1]*

*Diagnostic Criteria*. They include at least 10% clonal bone marrow plasma cells, serum, or urinary monoclonal protein.

Myeloma-related organ dysfunction (CRAB criteria) is as follows:

 hypercalcemia (serum calcium > 11.5 mg/dL (2.88 mmol/liter));
 renal insufficiency (serum creatinine > 2 mg/dL (177 umol/liter));
 anemia (hemoglobin < 10 g/dL or >2 g/dL below the lower limit of the normal range);
 bone disease (lytic lesions, severe osteopenia, or pathologic fracture).

*Staging*. The International Staging System is as follows:

 Stage I: serum B2-microglobulin < 3.5 mg/liter, serum albumin > 3.5 g/dL;
 Stage II: serum B2-microglobulin < 3.5 mg/liter plus serum albumin <3.5 g/dL or serum B2-microglobulin 3.5 to <5.5 mg/liter regardless of serum albumin level;
 Stage III: serum B2-microglobulin > 5.5 mg/liter.

Soft-tissue plasmacytomas in multiple myeloma can arise either through direct extension from skeletal tumors or via hematogenous metastatic spread [2, 4]. It is hypothesized that extramedullary spread may be due to decreased expression of adhesion molecules allowing plasma cells to escape bone marrow [4]. Liver infiltration by plasma cells seems to manifest in two distinct patterns: diffuse (sinusoidal, portal, or mixed) and nodular [11, 12]; however, the origin of plasma cells in liver is obscure. These cells could be from bone marrow or may have originated and proliferated locally from the reticuloendothelial sites [12]. Only observational data are available [2].

Myeloma patients generally have elevated liver enzymes, especially alkaline phosphatase due to heavy plasmacytic infiltration of hepatic tissue [6]. However, elevations of liver enzymes do not correlate with the presence of histologic lesion [12]. Observational studies have shown higher LDH, ALP, CRP, and IL-6 levels with extensive liver involvement in patients with multiple myeloma. However, one cannot simply hold multiple myeloma accountable for elevated liver enzymes in patients with multiple myeloma. The risk of liver dysfunction due to fatty infiltration, hepatocellular necrosis, hemosiderosis, or granulomata is not more increased in multiple myeloma than any other hematological disease [12].

Current research is actively exploring genetic abnormalities involved in extramedullary disease as it is associated with decreased overall survival and absence of novel agents specifically for extramedullary disease [4]. Certain translocations, t(14; 16) and t(14; 20), along with mutations of k-ras and deletions of 17p have already been linked with extramedullary disease involvement. Hopefully, with gene expression profiling, specific extramedullary disease genes can be identified that could become target for treatment modality [1, 16].

Currently, management guidelines do not revolve around extramedullary disease but are circled mostly around the age of the patient and disease activity. Patients under 65 years of age with active disease are started immediately on induction therapy with thalidomide, lenalidomide, or bortezomib plus hematopoietic stem-cell transplant. Patients between 65 and 75 years of age, based on comorbidities, are considered for reduced-dose intensity autologous transplantation. However, patients older than 75 years are rarely considered for stem-cell transplant and therapy dosage is usually reduced [1].
